# Incidence rates of resistant enterotoxigenic Escherichia coli in fresh vegetables and salads

**DOI:** 10.1099/acmi.0.000957.v3

**Published:** 2025-07-28

**Authors:** Carlos Ramón Vázquez-Quiñones, Monica Rincón-Guevara, Iván Natividad-Bonifacio, Carlos Vázquez-Salinas, Humberto González-Márquez

**Affiliations:** 1 Doctorado en Ciencias Biológicas y de la Salud, Universidad Autónoma Metropolitana, Unidad Iztapalapa, Iztapalapa, CDMX, Mexico; 2 Laboratorio Divisional de Espectrometría de Masas, División de Ciencias Biológicas y de la Salud, Universidad Autónoma Metropolitana, Unidad Iztapalapa, Iztapalapa, CDMX, Mexico; 3 Laboratorio de Microbiología Sanitaria, Departamento de Microbiología de la Escuela Nacional de Ciencias Biológicas, Instituto Politécnico Nacional, Carpio y Plan de Ayala s/n col. Sto. Tomas, Alcaldía, Miguel Hidalgo, CDMX, Mexico; 4 Laboratorio de Inocuidad Alimentaria, Departamento de Biotecnología, División de Ciencias Biológicas y de la Salud, Universidad Autónoma Metropolitana-Iztapalapa, San Rafael Atlixco 186, Leyes de Reforma 1ra Secc, Iztapalapa, CDMX, Mexico; 5 Laboratorio de Expresión Génica, Proteómica, Ciencias de la Salud, Iztapalapa, Universidad Autónoma Metropolitana, Unidad Iztapalapa, Av. San Rafael Atlixco 186, Leyes de Reforma 1ra Secc, Iztapalapa, CDMX, Mexico

**Keywords:** antimicrobial resistance, enterotoxigenic *Escherichia coli* (ETEC), *Escherichia coli*, fresh vegetables, incidence, matrix-assisted laser desorption/ionization-time of flight (MALDI-TOF), pathotypes

## Abstract

Diarrhoeal diseases remain a significant global health challenge, particularly in developing regions such as Africa, Asia and Latin America, where they are a leading cause of child mortality. Contaminated food, including raw or undercooked vegetables, is a major transmission route for diarrhoeal pathogens such as norovirus, *Campylobacter*, non-typhoid *Salmonella* and pathogenic *Escherichia coli*. This study aimed to assess the prevalence of enterotoxigenic * E. coli* (ETEC), a key diarrhoeal pathogen, in fresh produce and prepared salads in Mexico City. A total of 128 samples, including prepared salads (lettuce, carrots and tomatoes) and unprocessed coriander and lettuce, were analysed over 2 years using protocols from the Bacteriological Analytical Manual and the Official Mexican Standard (NOM) SSA 210. Genotyping was performed to detect ETEC-specific virulence genes encoding heat-stable and heat-labile enterotoxins (*st* and *lt*), respectively. ETEC was identified in 9.9% of the total samples, representing 51.56% of the confirmed *E. coli* isolates. Contamination rates varied by food type, with coriander showing the highest prevalence (78.78%), followed by lettuce (9.09%) and prepared salads from La Vicentina Market (9.09%) and La Purísima Market (3.03%). Genotyping revealed that 12.12% of the ETEC-positive samples carried both *st* and *lt* genes, while 33.3 and 54.6% carried only the *lt* or *st* gene, respectively. In lettuce samples, 9.09% were positive for ETEC, with 3.03% carrying the *lt* gene, 3.03% the *st* gene and 3.03% both genes. Similarly, in coriander, 21.21% were positive for the *lt* gene, 51.51% for the *st* gene and 6.06% for both genes. These findings highlight the widespread presence of ETEC in fresh produce sold in Mexico City, posing a significant public health risk, particularly given the increasing consumption of raw vegetables. The study provides the first reported data on ETEC contamination ratios in Mexico City, emphasizing the urgent need for improved food safety measures, including better hygiene practices during production, handling and preparation of fresh produce. This research underscores the importance of ongoing surveillance and preventive strategies to mitigate the risk of foodborne diarrhoeal diseases in urban populations.

## Data Summary

All data used are presented in supplementary materials. A total of 128 vegetable samples were collected between January 2018 and February 2020 from three markets in Mexico City (La Vicentina, La Purísima, and Central de Abastos) within the Iztapalapa municipality. From these samples, 334 isolates with *E. coli*-like morphology were obtained, of which 64 isolates were confirmed as *Escherichia coli* through molecular (*uid*A gene amplification) and biochemical methods. Of the confirmed *E. coli* isolates, 51.56% were identified as enterotoxigenic *E. coli* (ETEC) by amplification of the *st* and *lt* genes. The *st* gene was detected in 54.6% of ETEC isolates, the *lt* gene in 33.3%, and both genes in 12.2%. ETEC prevalence was highest in coriander (78.78%), followed by lettuce (9.09%) and mixed salads (12.82%). Antimicrobial susceptibility testing of 30 ETEC isolates revealed 100% resistance to cefotaxime and 97% to gentamicin. Multidrug resistance (MDR), defined as resistance to three or more antibiotic classes, was observed in several isolates, with two strains showing resistance to six of seven antibiotics tested. The highest sensitivity was recorded for amikacin (90%) and chloramphenicol (83%).

## Introduction

Diarrhoeal diseases are a severe health problem and one of the leading causes of child mortality, particularly in Africa, Asia and Latin American countries [1]. The World Health Organization estimates almost 1.7 billion episodes of diarrhoea in children globally each year, resulting in the fatality of nearly 450,000 infants under the age of 5 and another 50,000 children between 5 and 9 years old [2].

Diarrhoeal diseases are the most common manifestation of foodborne illness, with children being especially vulnerable [3]. In Mexico, intestinal illness represented the second leading cause of morbidity in children under 5 years old, according to the 2022 Annual of Morbidity from the Epidemiology General Management. Additionally, the Mexican General Directorate of Epidemiology bulletin reported over 600,000 cases of acute diarrhoeal illness (EDA) the following year, underscoring the public health significance of this issue [4]. Diarrhoea is often caused by consuming contaminated meat, raw or undercooked eggs, and improperly washed fruit and vegetables that harbour norovirus, *Campylobacter*, non-typhoid *Salmonella* and pathogenic *Escherichia coli* [3].

*E. coli* is a common inhabitant of the mammalian digestive tract and is typically transmitted via the faecal-oral route. Diarrhoeagenic *E. coli* harbours virulence factors that enable its adherence to the intestinal mucosa, leading to persistent infections, inflammation and lesions in the host and an immunologic reaction characteristic of each case. These characteristics have led to the classification of *E. coli* into six pathotypes: adherent invasive *E. coli*; diffusely adherent *E. coli*; enteroaggregative *E. coli* (EAEC); enteroinvasive *E. coli*; enteropathogenic *E. coli* (EPEC); enterotoxigenic *E. coli* (ETEC) and Shiga toxin-producing *E. coli* (STEC) [5,6].

A nationwide survey in Mexico indicated that *E. coli* pathotypes, especially ETEC and EAEC, show virulence determinants linked to severe gastroenteritis outbreaks [7,8]. Data on STEC are particularly critical. Outbreak investigations have confirmed that STEC, particularly non-O157 serotypes, contribute significantly to epidemic diarrhoea, often related to foodborne transmission [7]. The endemic nature of such infections is underscored by studies that identify *E. coli* strains with varying virulence profiles across different populations, indicating that certain strains are constantly circulating within the community, while others appear during specific outbreaks [9,10].

Several studies have documented the prevalence of *E. coli* pathotypes across Mexico. For example, research focusing on travellers to Cuernavaca revealed that food contamination, along with ambient temperature and rainfall, could significantly enhance the presence of various *E. coli* strains (ETEC and EPEC), contributing to traveller’s diarrhoea and affecting the incidence of diarrhoeal diseases caused by these pathogens [11].

Fruit, vegetables (especially fresh salads) and seasonings are among the foods that can be contaminated with one of these micro-organisms. Over the past 30 years, global *per capita* consumption of fresh vegetables has increased by 25%, with production rising 30% in the same period (from 30 to 60 million metric tonnes). Vegetables are commonly consumed both in establishments and on the streets. It is well known that vegetable consumption is associated with a rising number of illnesses due to bacterial contamination and poor hygienic practices during processing [12].

From a health perspective, it is crucial to document the prevalence of pathogens transmitted through vegetables better. While the per capita consumption of fresh vegetables in the form of salads in Mexico is unknown, the mean global consumption is estimated at 186 g day^–1^, with Central America at 56 g day^–1^ and Central Asia at up to 349 g day^–1^ [13].

Horticultural products can get contaminated at various points along their production chain, from crop growth conditions, including residual water usage for irrigation, to the point of sale and even during consumption. This contamination raises concerns about the increasing presence of pathogens such as *E. coli* producing broad-spectrum beta-lactamases (ESBL *E. coli*) [14,16]. Antimicrobial resistance among diverse micro-organisms is an escalating public health emergency due to the difficulties in treating these infections and the increasing prevalence of multi-antibiotic-resistant strains [17].

This study aims to identify the presence of *E. coli* pathotypes in fresh salads, lettuce and coriander sold or consumed in markets and on the streets in some regions of Mexico City and to determine the prevalence of resistance to the most common antibiotics used for the treatment of gastrointestinal illness in Mexico.

## Methods

### Sampling

Between January 2018 and February 2020, 128 samples of vegetable products were analysed. These samples included 32 samples of lettuce, cucumber and carrot salads obtained from the ‘La Vicentina’ market (EV1); 32 samples of cucumber, lettuce, carrot and beet salads obtained from the ‘La Purísima’ market (EV2); 32 samples of romaine lettuce; and 32 samples of coriander from the ‘Central de Abastos’ market. All collection sites were in the Iztapalapa municipality in Mexico City. Each sample weighed ~300 g and was transported in a hermetically sealed container at an approximate temperature of 10 °C. The time elapsed between collection and analysis was kept to under an hour to ensure the integrity and accuracy of the results.

### Microbiological analysis

For *E. coli* isolation and identification, the methodology recommended by the FDA’s Bacteriological Analytical Manual [18] and the NOM 210-SSa1-2014 was followed. All the samples in their original containers were chopped into small pieces. Afterwards, 10 g was placed in 90 ml of 0.1% peptone water (Difco^®^) for a 10^–1^ dilution. A series of ten-fold dilutions were prepared up to 10^−3^. For the Most Probable Number analysis (MPN) of faecal coliforms, a 3.3.3 series was used. Briefly, 1 ml of each dilution was placed in tubes containing 9 ml lactose broth with an inverted Durham tube. The tubes were incubated at 35+2 °C during 48+2 h. The tubes showing growth and gas production were inoculated by transferring 2–3 loops of inoculum onto *E. coli* broth (EC) (Difco^®^) containing an inverted Durham’s tube. They were incubated at 45.5+0.2 °C in an agitated water bath for 48 h. Biochemical tests for the identification of *E. coli* were performed.

### Molecular analysis

#### Bacterial DNA isolation and PCR

Presumptive *E. coli* was inoculated on capped test tubes containing 5 ml of brain heart infusion (BHI) broth and incubated at 37 °C for 24 h. Wizard Genomic DNA purification (PROMEGA) commercial kit was used following the manufacturer-recommended protocol.

#### Amplification of the target genes

Single PCR reaction was standardized for the amplification of the fragments of the target *E. coli* genes and for every pathotype. Reaction mix consisted of 12.5 µl Master Mix 2X (Thermo Scientific), 6.5 µl of nuclease-free water, 2 µl of forward primer, 2 µl of reverse primer (Table S1, available in the online Supplementary Material) and 2 µl of DNA in a total volume of 25 µl.

Conditions used for the *β*-glucuronidase (*uid*A) gene amplification were denaturalization at 94 °C 5 min^–1^ followed by 30 cycles at 55 °C 30 s^–1^, 72 °C 1 min^–1^ and a final extension at 72 °C 7 min^–1^. Amplification conditions for the plasmid-encoded toxin (*pet*) and the aggregative operon (*aggR*) (EAEC) genes and ETEC’s thermolabile and thermostable genes (*It* and *st*) included an initial denaturalization step at 94 °C 5 min^–1^, followed by 30 cycles at 94 °C 1 min^–1^, 52 °C 1 min^–1^, 72 °C 1 min^–1^ and a final extension at 72 °C 7 min^–1^. For the Shiga-like toxin 1 and 2 (*stx1* and *stx2*) (EHEC) genes, the intimin-encoding gene (*eaeA*) and the principal structural subunit of bundle-forming pili (*bfpA*) (EPEC) genes, an initial denaturalization was performed at 94 °C 5 min^–1^ followed by 30 cycles at 94 °C 1 min^–1^, 58 °C 1 min^–1^, 72 °C 1 min^–1^ and a final extension at 72 °C 7 min^–1^.

A Labnet MultiGene^™^ Gradient PCR Thermal Cycler (TC-9600G-230V, Staffordshire, UK) was used, and PCR amplification products were visualized by electrophoresis on a 1.5% agarose gel where 5 µl of each sample was mixed with 2 µl of green loading buffer (Jenna Bioscience^®^). Electrophoresis was performed using Tris-Glacial acetic acid-EDTA (TAE) buffer, applying 60 V current for 30 min. In brief, 100 bp molecular weight was used. Finally, the amplification products were visualized on a blue light transilluminator Model MBE-150 Major Science^®^. *E. coli* EAEC ATCC29552, EHEC ATCC 43895, EPEC ATCC 43887 and ETEC ATCC 35401 strains were used as positive controls and were donated by the Molecular Microbiology Laboratory of the Escuela Nacional de Ciencias Biológicas. The strains were maintained at −20 °C in BHI broth +10% glycerol until their use.

### Strain identification confirmation with the MALDI Biotyper^®^ system

*E. coli* confirmed isolates using biochemical identification were analysed by the MALDI Biotyper protocol from Bruker Daltonics on a mass spectrophotometer Autoflex Speed MALDI-TOF/TOF, using a *α*-Cyano-4-hydroxycinnamic acid matrix in 50% acetonitrile, 2.5% trifluoroacetic acid and Milli-Q^®^ water to a final concentration of 10 mg ml^−1^.

Each sample was processed as follows: 300 µl of Milli-Q water + culture was mixed with 900 µl of ethanol and centrifuged at 14,000 r.p.m. on an Eppendorf centrifuge, and the supernatant was discarded. Depending on the sample amount, the pellets were left to dry and resuspended in 5, 10 or more μl of 70% formic acid. This mixture was homogenized with a micropipette, and the same volume of acetonitrile was added and centrifuged one more time. Afterwards, 1 µl of the supernatant was placed on each well of a 98-sample stainless steel MALDI plate containing 1 µl of the matrix. Manufacturer’s directions were followed for the interpretation of the results.

### Antimicrobial susceptibility

For antimicrobial susceptibility, the methodology described by the Clinical and Laboratory Standards Institute (CLSI, M100 ED30: 2020) was followed. *E. coli* strains were inoculated on tubes containing 5 ml of trypticasein soy broth and incubated at 37 °C for 24 h. Afterwards, 100 µl of these pre-cultures were inoculated on tubes containing 5 ml of trypticase soy broth. They were incubated with agitation for 3 h until they reached turbidity like the MacFarland 0.5 nephelometer tube. Briefly, 1 ml of these cultures was spread onto a Mueller–Hinton agar plate where seven filter paper discs were placed on equidistant zones, each one impregnated with the following antibiotics: amikacin (30 µg), ampicillin (30 µg), cefotaxime (30 µg), ceftriaxone (30 µg), chloramphenicol (30 µg), gentamicin (10 µg) and sulphamethoxazole/trimethoprim (25 µg). Plaques were incubated at 37 °C for 24 h, and afterwards, the inhibition zones were measured as determined by the CLSI on M100 ED31: 2021. Each assay was performed in duplicate to ensure the precision of the results.

The antibiotic susceptibility assay results were compared with the zone diameter table for *Enterobacterales* of the M100 ED30: 2020 data from the CLSI (Table S2) [19].

## Results

### Microbiological analysis

From the 128 analysed samples, 334 isolates with morphological characteristics consistent with *E. coli* were obtained. Subsequently, 78 isolates were confirmed as *E. coli* through biochemical testing. These isolates were obtained from coriander samples (50%) and 26 from lettuce samples (33.33%). Ten isolates were obtained from fresh green salads containing cucumber and carrot (EV1) (12.82%), while only three isolates were obtained from the salads containing cucumber, carrot, lettuce and beetroot (3.85%) (Table 1).

**Table 1.** Identified and confirmed isolated strains by PCR and MALDI-TOF as *E. coli* isolated from vegetables and salads in Mexico City

Sample (type)	Sample (no.)	*E. coli* identification method			
		Morphology *E. coli*	Biochemistry *E. coli*	PCR (*uid*A+)	MALDI-TOF *E. coli*
Coriander	32	83	38	34	32
Lettuce	32	83	27	26	25
EV1	32	84	10	9	6
EV2	32	84	3	2	1
Total	128	334	78	71	64

Green salad 1 (EV1): cucumber, lettuce and carrot; green salad 2 (EV2): cucumber, carrot, lettuce and beetroot; *uid*A+: positive for *β*-glucuronidase gene.

These findings highlight the significant prevalence of *E. coli* in coriander and lettuce, with a notably lower occurrence in mixed salads. The variation in contamination rates among different vegetable products underscores the importance of targeted microbial assessments and interventions to ensure food safety in diverse market settings.

### Molecular analysis

**Fig. 1.**
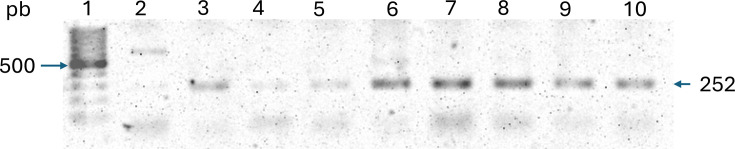
Gel electrophoresis used to identify *E. coli* utilizing the presence of the *uid*A gene for isolates from fresh salads, lettuce and coriander. Lane 1, 100 pb MW marker; Lane 2, negative control; Lane 3, ten confirmed isolates.

Molecular identification of the 78 presumptive *E. coli* isolates confirmed 71 by amplifying the *uid*A*+* gene (91.03%) (Fig. 1), indicating that seven isolates were incorrectly identified as *E. coli*. To further validate these results and ensure accurate species-level identification, we employed MALDI-TOF MS, a highly reliable technique for microbial identification based on protein profiles. MALDI-TOF analysis corroborated the presence of other micro-organisms within the coliform group that exhibited biochemical characteristics like *E. coli*. Specifically, the misidentified isolates were identified as *Klebsiella pneumoniae* (five isolates) and *Enterobacter cloacae* (two isolates). These isolates were consequently excluded from further analysis. The use of MALDI-TOF was critical in this study because it provided a high-resolution, rapid and accurate method for distinguishing *E. coli* from closely related species that share similar biochemical traits. This step ensured the reliability of our final dataset, confirming that out of the 78 presumptive isolates, 64 were indeed *E. coli*. The integration of molecular (*uid*A gene amplification) and proteomic (MALDI-TOF) approaches strengthened the robustness of our identification process and minimized the risk of misclassification (Table 1).

#### *E. coli* pathotype search

For the molecular identification of *E. coli* pathotypes, target genes were amplified in the 64 confirmed isolates. Of these, 51.56% were identified as ETEC. We did not detect the presence of Enterohaemorrhagic *E. coli* (EHEC), Enteroaggregative *E. coli* (EAEC) and/or Enteropathogenic *E. coli* (EPEC) pathotypes. The ETEC pathotype was determined by amplifying the *st* and *lt* genes (Fig. 1). Specifically, in 33.33% of the isolates, we successfully amplified the *lt* gene fragment. In 54.5%, we amplified only the *st* gene fragment. Both genes were found in 12.2% of the cases.

#### Antimicrobial susceptibility

Most isolated ETEC resisted at least two antibiotics. Predominant resistance was found for gentamicin (97%) and cefotaxime (100%). Less resistance was observed for ampicillin (50%), sulphamethoxazole/trimethoprim and chloramphenicol, which have a lower incidence of resistance (Table 2).

**Table 2.** Antimicrobial activity incidence shown by different ETEC strains isolated from several vegetables collected in Mexico City

Antibiotic	Resistance					Sensitivity				
	Lettuce	Coriander	EV1	EV2	Total	Lettuce	Coriander	EV1	EV2	Total
AK	0	2	1	0	3	2	22	1	2	27
AM	1	10	2	1	14	1	14	0	0	15
CFX	2	24	2	2	30	0	0	0	0	0
CTX	1	9	1	1	12	1	15	1	1	18
CL	0	4	0	1	5	2	20	2	1	25
GE	2	23	2	2	29	0	1	0	0	1
STX	0	12	1	0	13	2	12	1	2	17

Conversely, the ETEC strains exhibited greater sensitivity to amikacin (90%) and chloramphenicol (83%). Notably, three strains demonstrated resistance to both amikacin and gentamicin. Two strains were sensitive to amphenicols (chloramphenicol) and sulphonamides, while another was sensitive to amphenicols and *β*-lactams.

Multidrug resistance was evaluated according to the ReLAVRA protocols [20]. Table 3 shows the resistance profile of the 30 ETEC isolates evaluated with the 7 antibiotics. Notably, two strains isolated from coriander exhibited extended resistance to six of seven tested antibiotics, remaining sensitive to amikacin. Among the multi-resistant strains of five antibiotics, the sensitivity incidence was lower for amikacin (37.5%), chloramphenicol (25%), sulphamethoxazole/trimethoprim (25%) and ampicillin (17.5%) (Table 3).

**Table 3.** Multi-resistance in identified ETEC strains isolated from several vegetables in Mexico City

	R0	R1	R2	R3	R4	R5	R6	R7
Coriander (*n*=24)	0 (0)	0 (0)	6 (25)	7 (29)	6 (25)	3 (13)	2 (8)	0 (0)
EV1 (*n*=2)	0 (0)	0 (0)	–	–	1 (50)	1 (50)	–	0 (0)
EV2 (*n*=2)	0 (0)	0 (0)	–	1 (50)	–	1 (50)	–	0 (0)
Lettuce (*n*=2)	0 (0)	0 (0)	–	2 (100)	–	–	–	0 (0)
Total (*n*=30)	0 (0)	0 (0)	6 (20)	10 (33.3)	7 (23.3)	5 (16.7)	2 (6.7)	0 (0)

R0 – Sensitive to all antibiotics tested in this study; R1, R2, R3, R4, R5, R6 and R7 – Resistant to 1, 2, 3, 4, 5, 6 and 7 antibiotics tested, respectively.

## Discussion

Various outbreaks of foodborne diseases have been associated with consuming contaminated fresh vegetables. Information regarding this issue is continuously updated in Europe, North America, Australia and New Zealand due to a regulated epidemiological vigilance system. However, developing countries in Latin America face the need for more data regarding food safety, the difficulty accessing clean water, scarce agricultural infrastructure and limitations in implementing good farming practices, which present persistent challenges that prevent adequate control of gastrointestinal illnesses [21].

Vegetables can get contaminated during farming, collection, commercialization and consumption. Vegetables consumed raw without adequate sanitization can potentially cause gastrointestinal illnesses. This highlights the importance of ensuring food is not contaminated with micro-organisms, their toxins or any other physical or chemical pollutant. Crops are often watered with wastewater, and farming soil is usually fertilized with untreated organic compost [22,24]. In this work, we found the presence of ETEC in 78.78% of the coriander samples, with 21.21% of them amplifying the *lt* gene, 51.51% the *st* gene and 6.06% both genes.

For lettuce samples, ETEC presence was detected in 9.09% of them, with 33.33% of the strains showing amplification of the *lt* gene, 33.33% of the *st* gene and 33.33% of both genes (*lt/st*). Currently, there are no data on this issue in Mexico City. ETEC pathotype presence has been reported in the country on alfalfa sprouts (2%) [25]; beetroot juice (2%) [26]; carrot juice (1.4%) [27]; round and ‘guaje’ tomato varieties (3–4%) [7]; on a dairy product (1.78%) [28]; and on coriander (2%) [29].

Diarrhoeic sickness caused by ETEC has been reported in South Asia (27%), Latin America (22%) and Africa (North and East) (12%) [30]. In Bangladesh, diarrhoeagenic *E. coli* was responsible for 34% of diarrhoeic cases in adults and 90% in infants, with ETEC being the prevalent strain in 23% of the samples. Likewise, ETEC and EPEC have been reported in surface waters [15,31]. Different types of diarrhoeagenic *E. coli* have been identified in Japan as the most frequent enteropathogenic agent, with 10% in Iran and 11% in Finland. In Thailand and Vietnam, ETEC was the pathotype causing diarrhoea in 5.8 and 4% of the reported cases, respectively [15,32, 33].

Regarding the presence of the *lt* (487 bp) and *st* (322 bp) genes, Table S4 shows that in 4 of the strains (12.12%), both gene fragments (*st*/*lt*) were amplified; in 11 strains (33.3%), only the *lt* gene was amplified; and in 18 strains (54.6%), only the *st* gene was amplified. So far, no data related to the presence of both target genes on ETEC in Mexico City have been obtained. Only the *st* gene was amplified in strains originating from beetroot juice [26] and unpasteurized carrot juice [27].

The importance of the presence of both genes on ETEC lies in the fact that the *lt* toxin activates the secretion of Cl^-^ mediated by cystic fibrosis transmembrane conductance regulator, increasing cAMP. In contrast, the *st* toxin inhibits NHE3 activity, deactivating the trafficking of the transporter [34,35]. A combination of both toxins could be crucial for developing traveller’s diarrhoea. Notably, 10–14% of patients with traveller’s diarrhoea after travelling to Latin America, Africa or Asia show irritable colon symptoms [36].

Regarding antibiotic sensitivity, we found that 100% of the isolated strains were resistant to cefotaxime and 97% to gentamicin. In total, 4 strains showed resistance to *β*-lactams, aminoglycosides, amphenicols and sulphonamides; 9 strains displayed multi-resistance to *β*-lactams, amphenicols and aminoglycosides; 1 strain exhibited resistance to *β*-lactams, amphenicols and sulphonamides; and 17 strains had heterogeneous resistance.

This research showed the specific sensitivity of ETECs to some antibiotics: 90% for amikacin (27/30), 60% for ceftriaxone and 3.3% for chloramphenicol. Two strains were considered sensitive to amphenicols and sulphonamides, and one exhibited sensitivity to amphenicols and *β*-lactams. This last data are comparable to a review from the African Continent reporting * E. coli* with 53% sensitivity to gentamicin, 49.3% to ciprofloxacin and 47.2% to cotrimoxazole (sulphamethoxazole/trimethoprim) [37].

A 2016 report found ETEC strains with multi-resistance to amikacin, gentamicin and sulphamethoxazole/trimethoprim [29]. In 2018, multi-resistance to amikacin, erythromycin, gentamicin, colistin, kanamycin, ceftriaxone and compounds such as amoxicillin/clavulanic acid or sulphamethoxazole/trimethoprim, among others, was reported in fresh cheese [37]. Comparatively, in our study, two ETEC strains showed multi-resistance to six different antibiotics, including ampicillin and gentamicin, but were sensitive to amikacin.

No published information addresses the predominance of genes (*st* or *lt*), suggesting a gene–resistance correlation. However, our results indicate that from the strains resistant to four groups of antibiotics and isolated from coriander samples, 75% showed expression of the *st* gene during molecular identification. In contrast, only 25% expressed the *lt* gene. Similarly, from the strains isolated from coriander and showing resistance to three groups of antibiotics, 71.5% expressed the *st* gene, and only 14.3% expressed the *lt* gene.

In this study, 4 of the 30 ETEC strains showed antimicrobial resistance to all the antibiotics tested (*β*-lactams, aminoglycosides, amphenicols and sulphonamides). Two studies focused on multi-resistance reported *E. coli* resistance to different antibiotics. The first mentions resistance to erythromycin, amoxicillin, tetracycline, cotrimoxazole and chloramphenicol, among others. In 2019, this micro-organism showed resistance to gentamicin, cefuroxime, cefotaxime, carbapenems and quinolones. These two studies were conducted on uropathogenic *E. coli* [38,39].

Similarly, in our study, multi-resistance to gentamicin, cefuroxime, cefotaxime, amphenicols (chloramphenicol) and sulphonamides (sulphamethoxazole/trimethoprim) was related to the gastrointestinal tract. Additionally, 17 strains showed heterogeneous resistance to two antibiotics of any group. Our data suggest no direct relationship between the ETEC strains and the vegetables. Thus, a direct relationship between vegetable presence, pathogen presence and multi-resistance cannot be confirmed, posing a threat to the consumer’s health.

Lifestyle modifications have significantly changed the consumption of ready-to-eat foods such as salads, increasing market demand. The presence of ETEC in fresh salads, lettuce and coriander sold and consumed in markets or on the streets in some areas of Mexico City suggests that this bacterium could be linked to the increasing number of gastrointestinal diseases.

This study shows that ETEC strains in the analysed vegetable products contain genes coding for toxins and exhibit antibiotic resistance, proving their pathogenic capability. Their presence on contaminated Ready-To-Eat (RTE) salads or in vegetables such as lettuce and coriander poses an actual risk for consumers.

## Recommendations and future research

Vegetables can get contaminated during farming, collection, commercialization and consumption. This means that vegetables consumed raw without proper sanitization can cause gastrointestinal illnesses, highlighting the importance of ensuring that our food is not contaminated with micro-organisms or toxins or even with physical or chemical pollutants. This problem is unsurprising because crops are often watered with wastewater, and the soil is fertilized with untreated organic compost. Additionally, coriander, a prevalent ingredient in dishes or salsas in Mexican cuisine, poses a security risk to consumers due to the lack of hygiene and sanitization applied to its handling. It is common for people to store it in buckets filled with non-drinking water all day long, making proper handling and food preparation critical for safety.

Consuming food prepared and sold on the streets or in open markets is risky, as it can contain harmful micro-organisms, posing a substantial health risk to consumers of all ages and social classes. The presence of ETEC in fresh salads, lettuce and coriander sold or consumed in markets or on the streets in some areas of Mexico City, combined with lifestyle changes that have generated the demand for vegetables, suggests that this bacterium could be related to the increasing number of gastrointestinal diseases.

## Supplementary material

10.1099/acmi.0.000957.v3 Uncited Supplementary Material 1.

## Abbreviations

BHI: brain heart infusion
CLSI: Clinical and Laboratory Standards Institute
EAEC: enteroaggregative *Escherichia coli*
EPEC: enteropathogenic *Escherichia coli*
ETEC: enterotoxigenic *Escherichia coli*
STEC: Shiga toxin-producing *Escherichia coli*
