# Supplementary material for: Incidence rates of resistant enterotoxigenic Escherichia coli in fresh vegetables and salads
Source: Access Microbiol. 2025 Jul 28;7(7):000957.v3. doi: 10.1099/acmi.0.000957.v3 (PMC12303537; doi:10.1099/acmi.0.000957.v3)
Supplement: Uncited Supplementary Material 1. [file acmi-7-00957-s001.pdf]

SUPPLEMENTARY MATERIALS

Table S1. Primer sequence for the target genes of different *Escherichia coli* pathotypes, amplicon size, and Tm referred to in other references.

Pathotype	Gene	Primer	Size	Reference
E. coli	*uidA* F	GCGAAAACTGTGGAATTGGG	252	(13)
	uidA R	TGATGCTCCATAACTTCCTG		
EPEC	*eaeA* F	TGAACTACCGTCAAAGTTAT	206	Designed for this study
	eaeA R	ATGACAAGGCATTAAATTAT		
	bfpA F	AAGAAATACGAAAAAGGTCT	232	Designed for this study
	bfpA R	GAATTGCAGATGTGTTAAGT		
EHEC	*stx1* F	CAGAGGAAGGGCGGTTTAAT	224	Designed for this study
	stx1 R	ATGGCGATTTATCTGCATCC		
	stx2 F	TCGCCAGTTATCTGACATTCTG	178	Designed for this study
	stx2 R	GCAATGTGCTTCCGGAGTAT		
EAEC	*aggR* F	CGGCTGTAAGCTTCTTTTCC	310	Designed for this study
	aggR R	GTCAAGAATTGTTTTGGTGTTATGC		
	pet F	GTGATTATGCGGGCTGATCT	248	Designed for this study
	pet R	TCCTGATCCTCTTTCGTGCT		
ETEC	lt F	GGCGACAGATTATACCGTGC	450	Tamayo-Legorreta *et al.*, 2021
	lt R	CGGTCTCTATATTCCCTGTT		
	st F	CTGTATTGTCTTTTTCACCT	322	(14)
	st R	GCACCCGGTACAAGCAGGAT		

SOURCE: (15-20).

Table S2. Antibiotic data and inhibition zone diameter for Enterobacterales were used to determine the ETEC strains' sensitivity.

Antibiotic	Abbreviation	Units (µg)	Sensitive	Intermediate	Resistant
Amikacin	AK	30	≥ 17	15-16	≤ 14
Ampicillin	AM	30	≥ 17	14–16	≤ 13
Cefotaxime	CFX	30g	≥ 26	23-25	≤ 22
Ceftriaxone	CTX	30	≥ 23	20-22	≤ 19
Chloramphenicol	CL	30	≥ 18	13-17	≤ 12
Gentamicin	GE	10	≥ 15	13-14	≤ 12
Sulfamethoxazole/ Trimethoprim	STX	25	≥ 16	11-159	≤ 10

Measure units in the inhibition zones are mm.

Table S3. MALDI-TOF/TOF confirmation of ETEC isolates in vegetable and salads.

Isolate	Date/sample	Origin	Biochemical confirmation	MALDI TOF identification
1	MAY 18	Fresh salad 1	*Escherichia coli*	*Escherichia coli*
2	MAY 18	Fresh salad 1	*Escherichia coli*	*Escherichia coli*
3	MAY 18	Lettuce	*Escherichia coli*	*Enterobacter cloacae*
4	MAY 18	Lettuce	*Escherichia coli*	*Escherichia coli*
5	MAY 18	Fresh salad 2	*Escherichia coli*	*Escherichia coli*
6	AUG 18	Fresh salad 1	*Escherichia coli*	*Escherichia coli*
7	AUG 18	Coriander	*Escherichia coli*	*Klebsiella Pneumoniae*
8	SEP 18	Coriander	*Escherichia coli*	*Escherichia coli*
9	SEP 18	Coriander	*Escherichia coli*	*Escherichia coli*
10	SEP 18	Coriander	*Escherichia coli*	*Escherichia coli*
11	SEP 18	Coriander	*Escherichia coli*	*Escherichia coli*
12	SEP 18	Coriander	*Escherichia coli*	*Escherichia coli*
13	SEP 18	Coriander	*Escherichia coli*	*Escherichia coli*
14	SEP 18	Coriander	*Escherichia coli*	*Escherichia coli*
15	SEP 18	Coriander	*Escherichia coli*	*Escherichia coli*
16	SEP 18	Coriander	*Escherichia coli*	*Escherichia coli*
17	SEP 18	Coriander	*Escherichia coli*	*Escherichia coli*
18	SEP 18	Coriander	*Escherichia coli*	*Escherichia coli*
19	SEP 18	Coriander	*Escherichia coli*	*Escherichia coli*
20	SEP 18	Coriander	*Escherichia coli*	*Escherichia coli*
21	SEP 18	Coriander	*Escherichia coli*	*Escherichia coli*
22	SEP 18	Coriander	*Escherichia coli*	*Escherichia coli*
23	SEP 18	Lettuce	*Escherichia coli*	*Escherichia coli*
24	SEP 18	Lettuce	*Escherichia coli*	*Escherichia coli*
25	SEP 18	Lettuce	*Escherichia coli*	*Escherichia coli*
26	SEP 18	Lettuce	*Escherichia coli*	*Escherichia coli*
27	SEP 18	Lettuce	*Escherichia coli*	*Escherichia coli*
28	SEP 18	Lettuce	*Escherichia coli*	*Escherichia coli*
29	SEP 18	Lettuce	*Escherichia coli*	*Escherichia coli*
30	SEP 18	Lettuce	*Escherichia coli*	*Escherichia coli*
31	SEP 18	Lettuce	*Escherichia coli*	*Escherichia coli*
32	SEP 18	Fresh salad 1	*Escherichia coli*	*Escherichia coli*
33	SEP 18	Fresh salad 1	*Escherichia coli*	*Klebsiella Pneumoniae*
34	SEP 18	Coriander	*Escherichia coli*	*Escherichia coli*
35	SEP 18	Coriander	*Escherichia coli*	*Enterobacter cloacae*
36	SEP 18	Coriander	*Escherichia coli*	*Escherichia coli*
37	SEP 18	Coriander	*Escherichia coli*	*Escherichia coli*
38	SEP 18	Coriander	*Escherichia coli*	*Escherichia coli*
39	SEP 18	Coriander	*Escherichia coli*	*Escherichia coli*
40	SEP 18	Lettuce	*Escherichia coli*	*Escherichia coli*
41	SEP 18	Lettuce	*Escherichia coli*	*Escherichia coli*
42	SEP 18	Lettuce	*Escherichia coli*	*Escherichia coli*

43	SEP 18	Lettuce	*Escherichia coli*	*Escherichia coli*
44	SEP 18	Lettuce	*Escherichia coli*	*Escherichia coli*
45	SEP 18	Lettuce	*Escherichia coli*	*Escherichia coli*
46	SEP 18	Lettuce	*Escherichia coli*	*Escherichia coli*
47	SEP 18	Lettuce	*Escherichia coli*	*Escherichia coli*
48	SEP 18	Lettuce	*Escherichia coli*	*Escherichia coli*
49	SEP 18	Lettuce	*Escherichia coli*	*Escherichia coli*
50	SEP 18	Lettuce	*Escherichia coli*	*Escherichia coli*
51	SEP 18	Lettuce	*Escherichia coli*	*Escherichia coli*
52	OCT 18	Coriander	*Escherichia coli*	*Escherichia coli*
53	OCT 18	Coriander	*Escherichia coli*	*Klebsiella Pneumoniae*
54	OCT 18	Lettuce	*Escherichia coli*	*Klebsiella Pneumoniae*
55	OCT 18	Lettuce	*Escherichia coli*	*Escherichia coli*
56	OCT 18	Lettuce	*Escherichia coli*	*Klebsiella Pneumoniae*
57	OCT 18	Fresh salad 1	*Escherichia coli*	*Klebsiella Pneumoniae*
58	OCT 18	Fresh salad 1	*Escherichia coli*	*Escherichia coli*
59	OCT 18	Fresh salad 1	*Escherichia coli*	*Escherichia coli*
60	OCT 18	Fresh salad 1	*Escherichia coli*	*Escherichia coli*
61	OCT 18	Fresh salad 1	*Escherichia coli*	*Escherichia coli*
62	JUL 19	Coriander	*Escherichia coli*	*Escherichia coli*
63	JUL 19	Coriander	*Escherichia coli*	*Escherichia coli*
64	JUL 19	Coriander	*Escherichia coli*	*Escherichia coli*
65	JUL 19	Coriander	*Escherichia coli*	*Escherichia coli*
66	JUL 19	Lettuce	*Escherichia coli*	*Escherichia coli*
67	JUL 19	Fresh salad 2	*Escherichia coli*	*Klebsiella Pneumoniae*
68	JUL 19	Fresh salad 2	*Escherichia coli*	*Escherichia coli*
69	AGO 19	Coriander	*Escherichia coli*	*Escherichia coli*
70	AGO 19	Coriander	*Escherichia coli*	*Escherichia coli*
71	AGO 19	Coriander	*Escherichia coli*	*Escherichia coli*
72	AGO 19	Coriander	*Escherichia coli*	*Escherichia coli*
73	AGO 19	Coriander	*Escherichia coli*	*Escherichia coli*
74	OCT 19	Coriander	*Escherichia coli*	*Escherichia coli*
75	OCT 19	Coriander	*Escherichia coli*	*Escherichia coli*
76	OCT 19	Coriander	*Escherichia coli*	*Escherichia coli*
77	OCT 19	Coriander	*Escherichia coli*	*Escherichia coli*
78	OCT 19	Coriander	*Escherichia coli*	*Escherichia coli*

Table S4. Presence of *lt* and *st* genes in confirmed *E. coli* strains isolated from fresh salads collected in CDMX (Iztapalapa) between May-October 2018.

Sample Date	Strain	Origin	Gen		
			lt	*st*	*lt/st*
May-18	5	EV1			✓
Aug-18	6	Coriander		✓	
	10	Coriander		✓	
	11	Coriander		✓	
	12	Coriander		✓	
	14	Coriander		✓	
	16	Coriander		✓	
	17	Coriander		✓	
	18	Coriander		✓	
	26	Coriander		✓	
	28	Coriander		✓	
	30	Coriander		✓	
	41	Coriander		✓	
	42	Lettuce		✓	
	54	Lettuce			✓
	55	Lettuce	✓		
SEP-18	10	Coriander	✓		
	25	Coriander		✓	
	27	Coriander	✓		
SEP-18	9	Coriander	✓		
	10	Coriander	✓		
	13	Coriander	✓		
	19	Coriander	✓		
	27	Coriander		✓	
	28	Coriander		✓	
	40	Coriander		✓	
	43	Coriander	✓		
OCT-18	4	EV1	✓		
	5	EV1	✓		
	37	EV2	✓		
	53	Coriander			✓
	55	Coriander		✓	
	60	Coriander			✓
Total	33		11	18	4
